# P19 Cells as a Model for Studying the Circadian Clock in Stem Cells before and after Cell Differentiation

**DOI:** 10.5334/jcr.157

**Published:** 2018-05-18

**Authors:** Abdullah Mashhour, Zainab Al Mansour, Al Shaima Al Hallaj, Rizwan Ali, Thadeo Trivilegio, Mohamed Boudjelal

**Affiliations:** 1 Medical Core Facility and Research Platforms, King Abdullah International Medical Research Center/King Saud bin Abdulaziz University for Health Sciences, Ministry of National Guard Health Affairs, Riyadh 11426, SA

**Keywords:** Circadian clock, molecular clock, stem cells, P19 cells, neurons

## Abstract

In mammals, circadian rhythmicity is sustained via a transcriptional/translational feedback loop referred to as the canonical molecular circadian clock. Circadian rhythm is absent in undifferentiated embryonic stem cells; it begins only after differentiation. We used pluripotent P19 embryonal carcinoma stem cells to check the biological clock before and after differentiation into neurons using retinoic acid. We show that the central clock genes *ARNTL (Bmal), Per2* and *Per3*, and the peripheral clock genes *Rev-erb-α* and *ROR-α*, oscillate before and after differentiation, as does the expression of the neuronal differentiation markers *Hes5*, β-3-tubulin (Tubb3) and *Stra13*, but not *Neurod1*. Furthermore, the known clock-modulating compounds ERK, EGFR, Pi3K, p38, DNA methylation and Sirtiun inhibitors, in addition to *Rev-erb-α* ligands, modulate the expression of central and peripheral clock genes. Interestingly Sirtinol, Sirt1 and Sirt2 inhibitors had the greatest significant effect on the expression of clock genes, and increased *Hes5* and *Tubb3* expression during neuronal differentiation. Our findings reveal a new frontier of circadian clock research in stem cells: contrary to what has been published previously, we have shown the clock to be functional and to oscillate, even in undifferentiated stem cells. Modulating the expression of clock genes using small molecules could affect stem cell differentiation.

## Introduction

The circadian rhythm is the physiological and behavioral about-24-hour cycle that occurs in most organisms. This includes the sleep and wake cycle, along with other factors such as hormonal levels, eating, drinking, energy metabolism, cell division, post-transcriptional regulation and regulation of the endocrine system. It is well known that external cues can influence this cycle; the best example of this is daylight, which – for most of us – is responsible for resetting this cycle [1]. In mammals, circadian rhythmicity is sustained via a transcriptional/translational feedback loop frequently referred to as the canonical molecular circadian clock. The positive elements *ARNTL (Bmal)* and Clock heterodimerize, and in doing so initiate transcription of the negative Period elements (*Per1, 2*, and *3*) and Cryptochromes (*Cry1* and *2*) through binding to E-box elements within the promoter regions of these negative elements. Per and Cry proteins heterodimerize and re-enter the master circadian clock, located in the suprachiasmatic nucleus (SCN) to prevent interactions between the positive elements clock and *ARNTL (Bmal)* [2].

An additional feedback loop, which includes two nuclear receptors, the orphan nuclear receptor, *Rev-erb*-α, and retinoic acid-related orphan receptor (*ROR*-α), helps to improve the robustness of the previous feedback loop. The nuclear receptors *Rev-erb*-α and *ROR*-α compete with each other to bind to the *ARNTL (Bmal)* promoter *ROR* element (*RORE*)/*RevRE* site, and then activate the transcription process [3].

Unlike differentiated cells, it is well known that embryonic stem cells (ESCs) have no circadian rhythms in gene expression when they are in the undifferentiated stage. Circadian rhythms begin during the differentiation of these cells [4,5,11]. Embryonal carcinoma (EC) stem cells are pluripotent, and have the ability to differentiate into any of the three primary germ layers: the endoderm, mesoderm and ectoderm [9,10]. Independent groups have examined circadian rhythms in ESCs stably transfected with bioluminescent luciferase driven by either an *ARNTL (Bmal)* promoter or the promoter for the clock-controlled gene *mDbp* as a model. Results from these studies show that individual undifferentiated ESCs are not rhythmic with respect to *ARNTL (Bmal)* or *mDbp*, however they became rhythmic after synchronization with forskolin cells directed towards a neural fate. Together, this evidence shows that the canonical molecular clock is not rhythmic in ESCs, thus we can conclude that the clock is not functional this early in development [8,9].

P19 cells are pluripotent EC stem cells derived from pluripotent germ cell tumors called teratocarcinomas. P19 cells can differentiate into different types of cell, including neurons, astroglia and microglia, and this differentiation can be controlled by the addition of toxic drugs. For example, it is possible to drive P19 cells that are a few days post-aggregation to form neurons and glial cells by adding certain concentration of retinoic acid (RA) to the culturing media. There is a lack of circadian studies of neurogenesis in vitro; therefore, understanding the circadian regulation of adult neurogenesis can help to optimize the timing of therapeutic procedures for patients with neurodegenerative disorders [4,6,7]. For example, few studies have examined clock gene expression earlier in mammalian embryogenesis. As reviewed by Vallone and colleagues [5], the inception of molecular circadian rhythms in the brain arises just before birth, and this is followed by rhythms expressed by peripheral tissues [10]. Three to four days before parturition, electrical activity in the SCN, and postnatal rhythms of clock gene expression, are preceded by rhythmic glucose [1,2].

The P19 cell model system provides some important advantages over other cell models: these cells are highly susceptible to incorporate and express ectopic genes, they can easily grow in serum-supplemented media, and can differentiate in large quantities. These advantages make P19 cells a promising in vitro model to study cellular differentiation pathways, and the molecular mechanisms underlying the differentiation of pluripotent stem cells, including the molecular clock [12,13].

Although no previous studies have used P19 cells as a model to study the circadian clock, many groups have used these cells in other prospective studies. For example, P19 cells were used to study differences in mitochondrial physiology between differentiated and undifferentiated P19 cells, and to study the effects of compounds such as Sirtinol and EX-527 on the neuronal differentiation of P19 stem cells [14,15]. Interestingly, *Stra13*, an early gene induced by RA in P19 cells during neuronal differentiation, has been shown to be involved in the function of the molecular clock [16,17].

In this study, we used P19 cells as a cellular model system to examine oscillation of genes involved in the circadian clock before and after neuronal differentiation. Moreover, we investigated the effect of compounds, such as Sirt1 modulators, on the expression and oscillation of neuronal marker genes and neuronal differentiation. We provide the first report of the oscillation of circadian clock genes in mouse embryonic stem cells, i.e. P19 cells, and conclude that these cells are a good model system on which to test drugs affecting the clock genes.

## Materials and methods

### Cell culture

Mouse P19 embryonic carcinoma cells (ATCC) were cultured in a 175-ml flask (Corning) using Dulbeccos’s Modification of Eagles Medium (DMEM) supplemented with 10% fetal bovine serum (FBS), 5% non-essential amino acid (NEAA), and 5% penicillin–streptomycin (Pen–Strip) and L-glutamine (all from Gibco). Cells were then left to grow in a cell culture incubator with 5% CO_2_ at 37°C until confluent.

### Differentiation of P19 cells by retinoic acid (RA)

For neurogenesis differentiation, confluent P19 cells were seeded at a density of 10^5^ cells/ml in 90-mm Petri dishes, and then incubated with 1 µM RA (TOCRIC) for 4 days. Media were changed once after the second day. The aggregate was then dissociated into single cells by treatment with non-enzymatic cell dissociation solution (celldiss; UFC Biotech). After washing cells with phosphate buffered saline (PBS; Gibco) to remove remaining RA and trypsin, they were seeded in tissue culture dishes at a density of 2 × 10^6^ cells/ml using DMEM supplemented with 10% FBS, 5% NEAA, 5% Pen-Strip, and L-glutamine, and 1 µM RA. After 48 hours, the culture media were replenished once, and dishes incubated for another 2 days to form neurons.

For the circadian studies, cells were cultured in six-well plates at 80% confluency, either as differentiated or undifferentiated neurons, at 37°C and 5% CO_2_. Cells were then synchronized using temperature changes by first transferring cells into an incubator at 32°C/5% CO_2_ for 12 hours, then the temperature was changed to 37°C for 12 hours. Cells were then collected by scraping, and were centrifuged every 4 or 6 hours depending on the study. We collected the cells from one well per time point, and pellets were stored in a freezer at –80°C until required for extraction.

### Cell imaging

After seeding the two groups of P19 cells in 90-mm Petri dishes as described above, images of both the control cells and differentiated cells were taken using an EVOS FL Auto System (Life Technologies) using a 10 × (LPlanFL) objective.

### RNA extraction and RT-PCR

Total RNA was isolated from each collected sample using the Pure Link RNA Mini Kit (Ambion, Life Technologies), following the manufacturer’s instructions. Synthesis of cDNA was performed with 1 µg/µl of total RNA using a High Capacity cDNA reverse transcriptase kit (ABI). Reverse transcription polymerase chain reaction (RT-PCR) was performed using the Taqman gene expression kit (ABI), and specific primers (as described in Table 1) using the 7900HT Fast Real-Time PCR system (ABI). Negative controls (those without primers or templates) and with *HPRT1* (a housekeeping gene) were run together with the samples, and each sample was run in duplicate. Copy numbers of tested genes were normalized to *HPRT1* (as an internal control) at different time points.

**Table 1:** Real time PCR Primers.

Gene	Species	Genebank accession	Amplicon length

Per2	Mouse	NM_011066.3	73
HPRT	Mouse	NM_013556.2	131
Reverba	Mouse	NM_145434.4	62
BMAL	Mouse	NM_001243048.1	87
PER3	Mouse	NM_001289877.1	73
RoRa	Mouse	NM_001289916.1	68
STRA13	Mouse	NM_016665.2	65
TuBB	Mouse	NM_023279.217	58
Hes 5	Mouse	NM_010419.412	73
Neurod1	Mouse	NM_010894.224	94

### Protein extraction and immunoblotting

Cell pellets intended for protein extraction were suspended in 100 µl T-PER tissue protein extraction buffer (Thermo); extraction buffer was supplemented with 10 µl protease inhibitor (Sigma–Aldrich). Cell samples were vortexed and then incubated on ice for 20 minutes. After incubation, samples were centrifuged at 4°C for 10 minutes at 14,000 rpm. The supernatants were then collected and protein concentrations were determined using the Qubit protein assay kit (Molecular Probes, Life Technologies) and the Qubit 2.0 Fluorometer System (Invitrogen, Life Technologies). All gel electrophoresis for immunoblotting was performed using Bio-Rad systems and buffers according to the manufacturer’s instructions. Lysates corresponding to equal amounts of proteins (50 µg) were supplemented with 2 × Laemmli sample buffer (Bio-Rad) and heated at 95°C for 5 minutes. Then, equal amounts of samples were loaded into mini protein TGX gels (Bio-Rad) and resolved by gel electrophoresis using Tris/glycine/SDS 1 × running buffer (Bio-Rad). Protein standards (Bio-Rad) were also loaded into the gels for assessment of protein molecular weight. Subsequently, proteins were transferred onto polyvinylidene difluoride membrane (PVDF; Bio-Rad) using the Trans-Blot Turbo Transfer System (Bio-Rad) and 1 × Trans-Blot Turbo Transfer Buffer (Bio-Rad). After protein transfer, PVDF membranes were blocked for 1 hour with 0.1% Tween (Dako) dissolved in PBS (Gibco) supplemented with 5% bovine serum albumin (BSA; Bioworld) on a shaker, and then probed with the primary antibody (Table 2), which was diluted in 2% BSA in 0.1% PBST overnight with gentle shaking. PVDF membranes were subsequently washed 3 times for 15 minutes with 0.1% PBST with gentle shaking, and then probed with species-specific secondary antibody (Table 2) diluted in 2% BSA for 1 hour at room temperature with gentle shaking. After probing, PVDF membranes were washed 3 times for 5 minutes with 0.1% PBST. Proteins were visualized using the Clarity Western ECL Substrate Kit and the ChemiDoc MP Imaging System (both from Bio-rad) according to the manufacturer’s instructions.

**Table 2:** Primary and Secondary Antibodies used in this study.

Antibody	Source	Dilution	Company	Cat. Num.

**Primary Ab**				

Beta-Actin	Mouse	1:1000	Cell Signalling	3700S
Beta-3-Tubulin	Mouse	1:1000	Cell Signalling	4466S
SEAA1	Mouse	1:1000	Cell Signalling	4744S
**Secondary Ab**				

Goat-Anti-Mouse	Mouse	1:3000	Bio-Rad	170–6516

### Immunocytochemistry

P19 cells were seeded in a 12-well plate (Corning, USA) and treated with 1 µl and 10 µl of RA for 8 days. Cells were then fixed, permeabilized and incubated with the diluted antibody against mouse SSEA-1 (Cell Signaling) overnight at 4°C. Secondary FITC antibody (Life Technologies) was used to detect antibody fluorescence, and counter-staining was performed with Hoechst (Life Technologies) to detect the nuclei. Cells were imaged using the EVOS FL Auto system (Life Technologies).

### Statistical analysis

Statistical differences between clock gene expression in P19 cells and control samples were determined using Student’s *t*-test, assuming unequal variances. *P* values ≤ 0.05 were considered to be statistically significant.

To calculate the clock parameters, oscillation data were subjected to the online clock software Biodare 2 (https://biodare2.ed.ac.uk/) using Linear detrending. Parameters were calculated using Morfitt [18].

## Results

### Induction of P19 cells with RA towards neural differentiation

**Figure 1:**
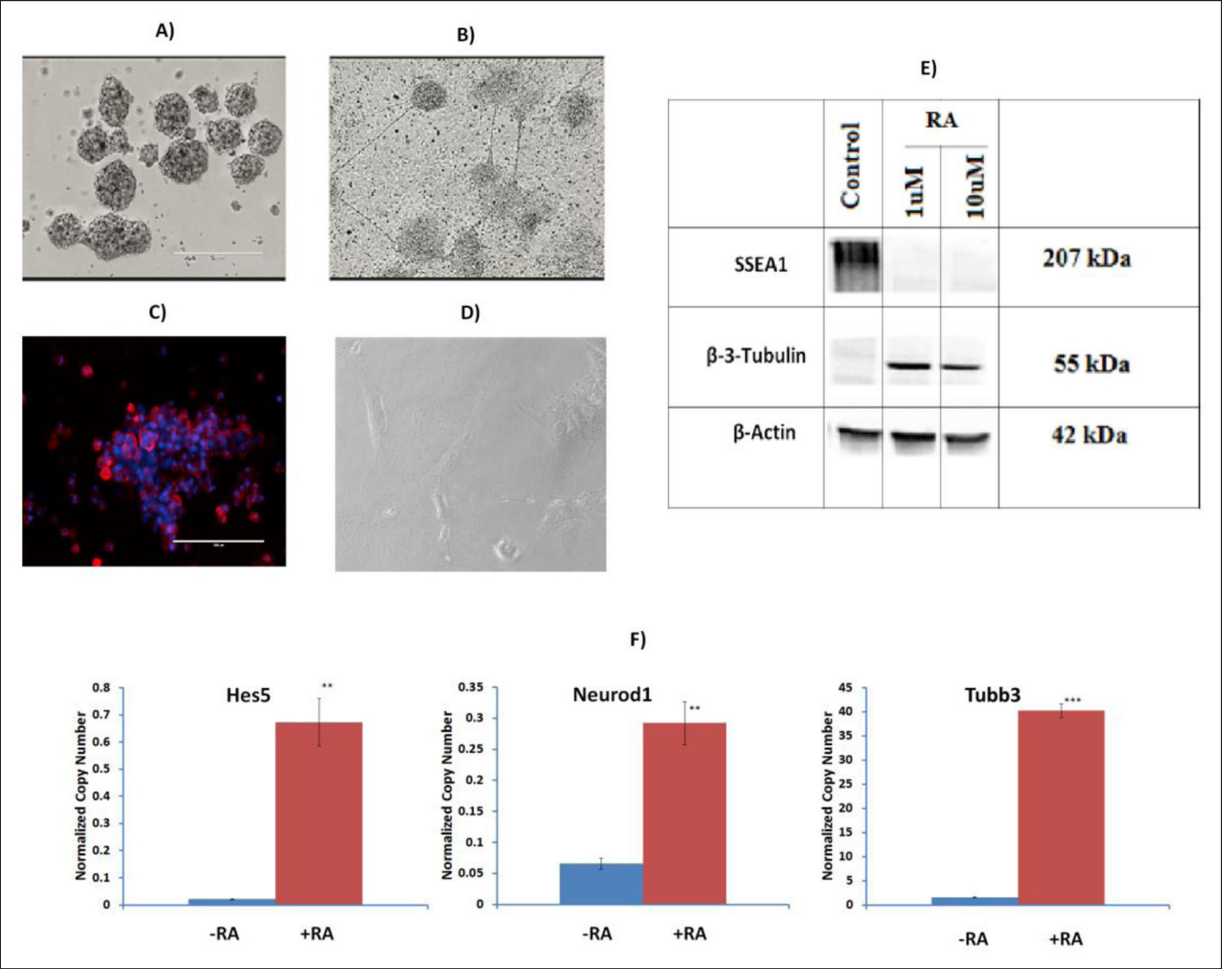
**Characterization of P19 cells used in the study.** P19 cells were grown in differentiation media containing 1 μM retinoic acid (RA). Images were taken using an Evos microscope after 4 days of aggregation **(A)**, and after P19 cells had formed neurons after differentiation treatment for 4 days **(B)**. To further validate the neuronal differentiation, immunoblot **(E)** analysis was performed to show expression levels of the stem cell marker SSEA1, and the neuron-specific marker β-3-tubulin in control (untreated) and differentiated (treated with 1 μM RA) P19 cells. β-actin was used as a control or housekeeping protein, and was run on a separate gel, as shown in the supplementary information. SSEA1 and β-3-tubulin were run together in another gel, as shown in the supplementary information. Immunostaining with SSAE1 was also performed **(C)** and **(D)**. The red color is SSEA1 staining (C), and the blue color in both (C) and (D) shows nuclear Hoechst staining. The gene expression levels of *Hes5, Neurod1* and *Tubb3* were analyzed in untreated and differentiated P19 cells **(F)**. Results show that the expression profile of the differentiated cells is visibly higher than that of the control.

P19 cells were induced towards neural differentiation by adding 1 µM RA as previously described by other researchers [19]. Images of control (untreated) cells (Figure 1A) and differentiated P19 cells (treated with RA, Figure 1B) were taken using the Evos microscope to demonstrate that neurons had already formed after four days. To further evaluate the neuronal differentiation in RA-treated P19 cells, the protein expression level of the neuronal marker Tubb3 was examined by immunoblot. As shown in Figure 1E, P19 cells treated with 1 µM RA showed clear expression of Tubb3, demonstrating the presence of neurons and that differentiation had been successful. To further validate the results, a mouse neural stem cell marker, SSEA1, was also tested using immunoblotting. Figure 1E shows the presence of this marker in untreated P19 cells, and its absence as cells differentiate into neurons, demonstrating that they are no longer in the pluripotent state. The housekeeping protein β-actin was used as a control in the immunoblot analysis, and its expression did not change. Immunostaining with SSEA1 as shown in Figure 1C and 1D, respectively, also shows clear staining of the SSEA1 antibody in undifferentiated P19 cells.

We also evaluated the expression profile of the well-known neural marker genes *Hes5, Neurod1*, and *Tubb3*, using reverse transcription quantitative polymerase chain reaction (qPCR) in both differentiated and undifferentiated P19 cells (Figure 1F). Our results show that the expression profile of the differentiated cells is visibly higher than that of the control cells.

### Expression of clock genes during P19 differentiation

**Figure 2:**
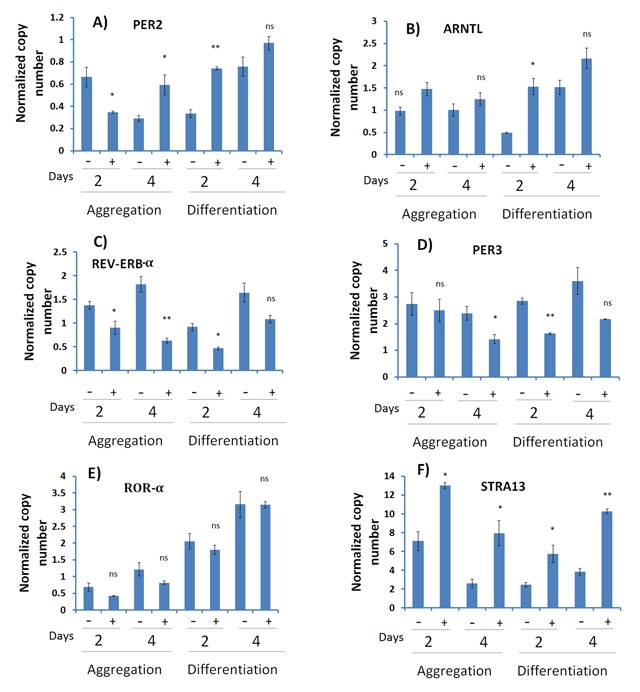
**Gene expression analysis of circadian clock genes in P19 embryonic stem cells.** P19 cells were grown as aggregates for 2 and 4 days, and then differentiated for 2 and 4 days in the absence (–) and presence (+) of 1 μM RA. Expression of *Per2* **(A)**, *ARNTL* (*Bmal*) **(B)**, *Rev-erb*-α **(C)**, *Per3* **(D)**, *ROR*-α **(E)**, and *Stra13* **(F)** were analyzed by qPCR. Data show that expression of clock genes is dynamic during the different phases of aggregation and differentiation by P19 cells, and the expression of *Stra13* increases in the presence of RA.

To investigate the expression of clock genes during the differentiation of P19 cells, RNA extracted from cells after 2 and 4 days of aggregation and differentiation phases was used for qPCR analysis. As evident from Figure 2, the aggregation and differentiation of P19 cells leads to differential expression of the clock genes. During the aggregation phase, the expression of *Per2* and *Per3* decreased in the presence and absence of RA on day 2 (Figure 2A and 2D). Expression of *Per3* continued to decrease on day 4 of aggregation, while *Per2* increased in the presence of RA. Expression of *Rev-erb*-α decreased in the presence of RA compared to its relative expression in the aggregate phase without RA (Figure 2C). In contrast, the expression of *ARNTL (Bmal)* and *ROR*-α increased in the presence of RA compared to the untreated aggregate both on day 2 and 4 (Figure 2B and 2E).

During the differentiation phase, the expression of *Per2, ARNTL (Bmal), Per3* and *ROR*-α increased after 2 and 4 days in the cells that were differentiated with RA compared to the control cells (Figure 2A, 2B, 2D, and 2E). In contrast, the expression of *Rev-erb*-α decreased at the same point in time (Figure 2C). These data demonstrate that the expression of the clock genes is dynamic during the different phases of P19 aggregation and differentiation, and indirectly indicate that these genes may be important in neural differentiation.

As a control, we also followed the expression of *Stra13*, an early RA-inducible gene and neuronal marker. We found that the expression of *Stra13* increases in the presence of RA in both aggregation and differentiation phases (Figure 2F).

### The circadian rhythm exists in differentiated and undifferentiated P19 cells

**Figure 3:**
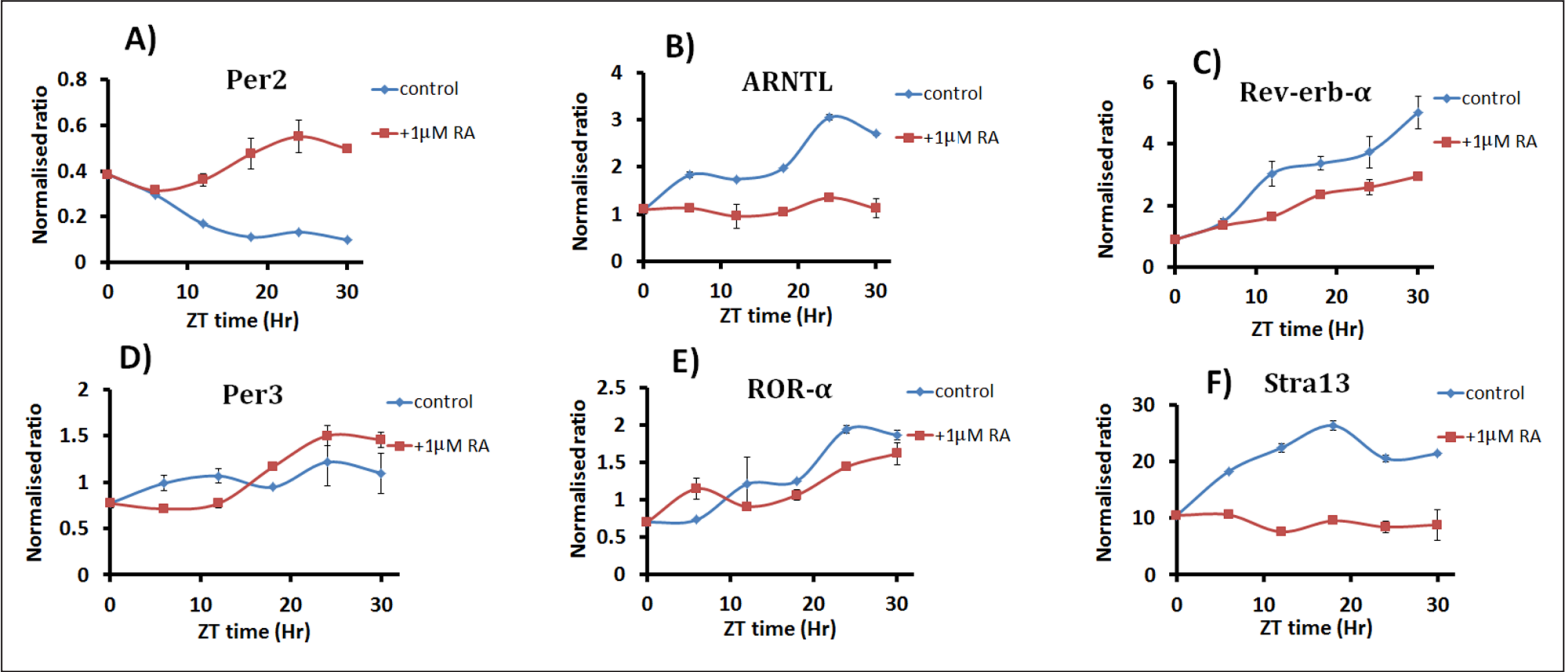
**Oscillation of the expression of circadian clock genes in P19 embryonic stem cells.** Blue line shows undifferentiated P19 cells (control); red line shows P19 cells differentiated by treatment with 1 μM retinoic acid (RA) for 4 days (differentiated). Cells were synchronized by temperature change and then collected after 0, 6, 12, 18, 24 and 30 hours for RNA extraction. Gene expression was analyzed by qPCR of *Per2* **(A)**, *ARNTL* (*Bmal*) **(B)**, *Rev-erb*-α **(C)**, *Per3* **(D)**, *ROR*-α **(E)** and *Stra13* **(F)** genes. Each time point was performed in triplicate. Data show means ± standard deviation. *Per2* and *Rev-erb*-α show weak oscillation patterns, while *ARNTL* (*Bmal*), *Per3* and *RoR*-α show significant oscillation patterns in both the control and differentiated cells. *Stra13* also shows a strong oscillation in the presence of RA.

To assess the rhythmicity of the expression of clock genes in both differentiated and undifferentiated P19 cells, we investigated the oscillation of *Per2, Rev-erb*-α, *ROR*-α, *ARNTL (Bmal), Per3*, and *Stra13*. To do this, we synchronized the cells by culturing them at 32°C/5% CO_2_ for 12 hours, and then switched them back to 37°C/5% CO_2_ for another 12 h. The cells were collected after every 6 hours for gene expression analysis as described in the methods. As shown in Figure 3, the clock genes do really oscillate in P19 cells, not only in the differentiated state, but in the undifferentiated state as well. For ease of comparison, we plotted the expression as the normalized ratio to the control (0 time point) for each gene. It is clear from the results that some genes show more robust oscillation than others. *Per2* (Figure 3A) and *Rev-erb*-α (Figure 3C) show weak oscillation patterns, while *ARNTL (Bmal)* (Figure 3B), *Per3* (Figure 3D), and *ROR*-α (Figure 3E) present clear and significant oscillation patterns in both the control and the differentiated cells. *Stra13* (Figure 3F), also shows a relatively strong oscillation pattern in the presence of RA.

To ensure that the observed clock gene oscillation was genuine, we ran the data through the detrending software Biodare 2 developed by Edward et al. [18]. The software detrented the data using the least formula, the linear detrending. As shown in Supplementary Figure 1, the detrented value of the gene expression data at different time points clearly shows that the expression oscillates. We also extracted clock parameters from the detrended data and, as summarized in Supplementary Figure 1E, the predicted periods for *ARNTL (Bmal), Per2* and *Per3* in undifferentiated cells were 33.46, 31.12, and 21.86 hours, respectively. In differentiated cells, the periods of both *ARNTL (Bmal)* and *Per3* become almost the same as that of *Per2*, ranging from 20.22 to 22.98 hours.

In undifferentiated P19 cells, the periods for *Rev-erb*-α and *ROR*-α were almost identical, ranging from 19–21 hours. However, in differentiated cells, the period for *Rev-erb*-α stayed almost the same, 22.98 hours, while the period for *ROR*-α period become elongated at 31.18 hours.

In terms of the phase, *ARNTL (Bmal)* and *Per3* were observed to oscillate in the opposite direction to that which was expected in undifferentiated P19 cells. However, in differentiated cells, *Per2* and *Per3* oscillated in the opposite direction to *ARNTL (Bmal)*.

**Figure 4:**
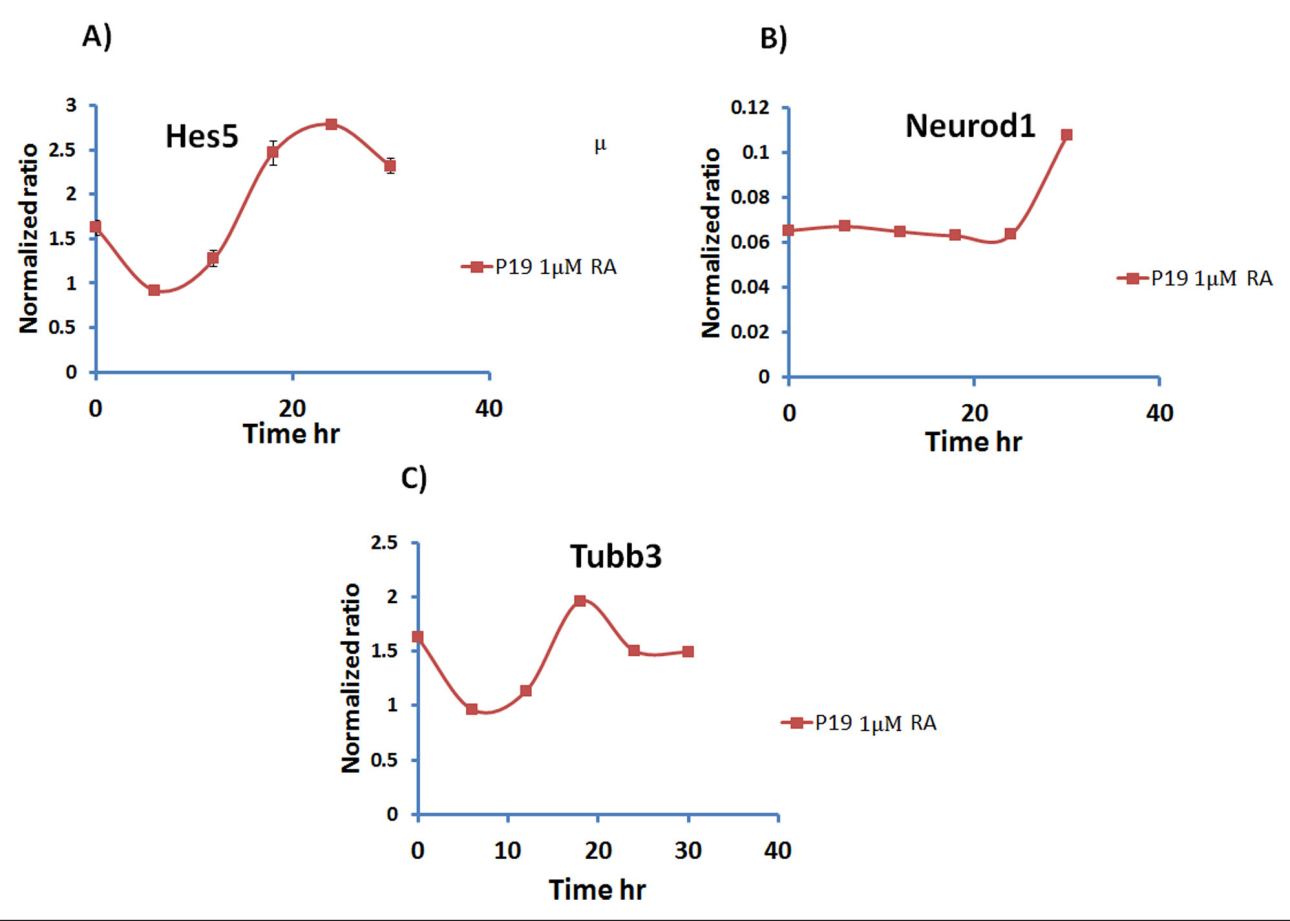
**Oscillation of neuronal differentiation markers in differentiated P19 embryonic stem cells.** P19 cells were grown for 4 days in the presence of 1 μM retinoic acid (RA). Cells were synchronized by temperature change and then collected after 0, 6, 12, 18, 24, and 30 hours before RNA extraction. Gene expression was analyzed by qPCR of *Hes5* **(A)**, *Neurod1* **(B)**, and *Tubb3* **(C)**. Each time point was performed in triplicate. Data shows means ± standard deviation. Differentiated P19 cells show clear oscillation of *Hes5* and *Tubb3*, while no oscillation of *Neurod1* was detected.

As well as the clock and *Stra13* genes, we examined the oscillation patterns of the neuronal marker genes *Hes5, Neurod1* and *Tubb3* after synchronization. Figure 4 shows clear oscillation patterns of *Hes5* (Figure 4A) and *Tubb3* (Figure 4C) in differentiated P19 cells, but no oscillation was seen in *Neurod1* (Figure 4B).

### Expression of clock genes in P19 cells treated with clock-associated drugs

To better assess the circadian clock in P19 cells, we tested several ligands of known factors associated with the biological clock: Sirtinol, an inhibitor of Sirt1 and Sirt2; EX 527, a selective inhibitor of Sirt1; SR 8278, a *Rev-erb*-α antagonist; and GSK 4112, a selective *Rev-erb*-α agonist. Of all the ligands tested, Sirt1, Sirt2 and *Rev-erb*-α have been shown to affect clock gene expression and oscillation in several cell types [23,24].

**Figure 5:**
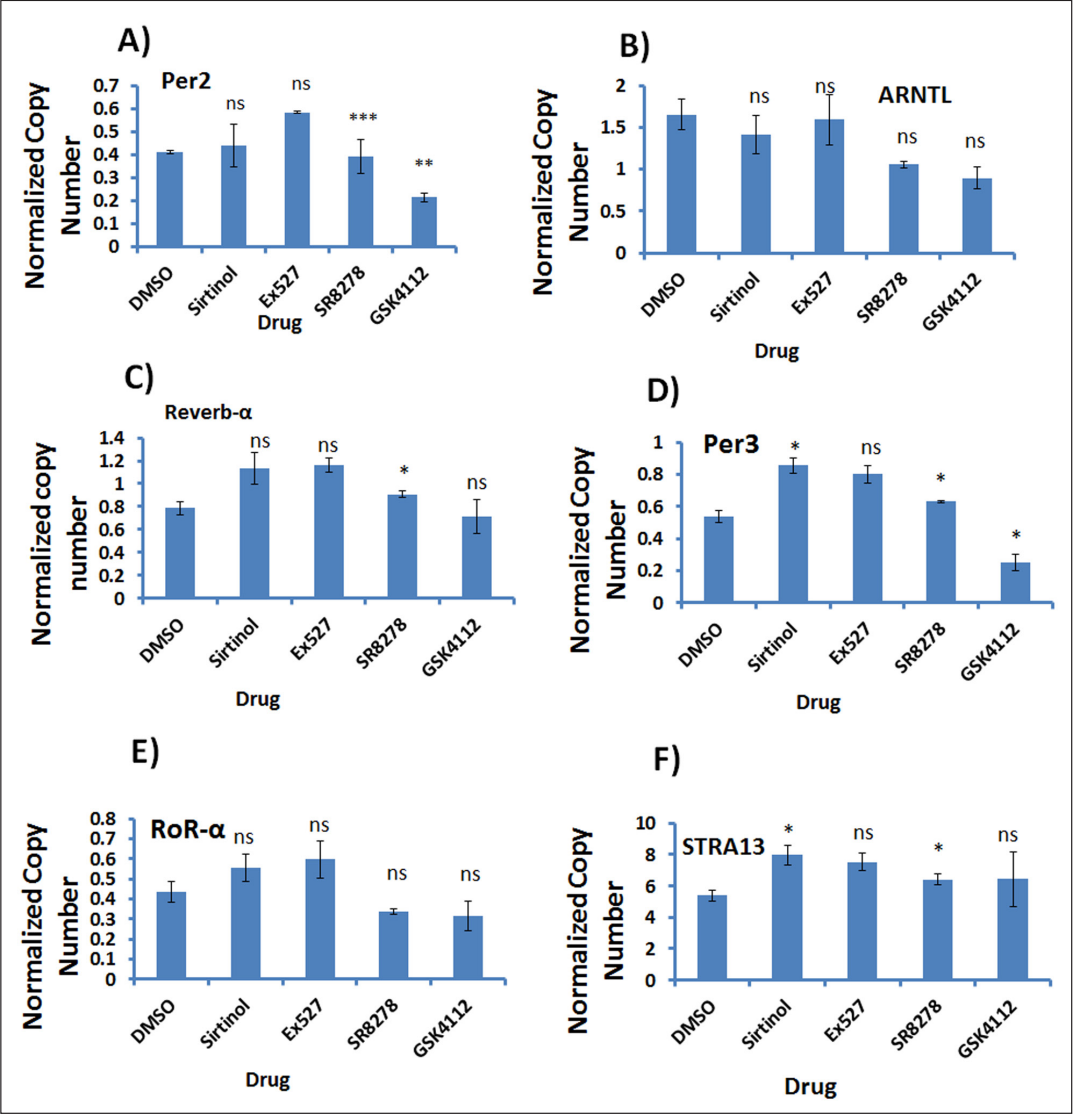
**Expression of clock genes in P19 cells treated with clock-associated drugs.** Using P19 cells, we tested several ligands of known factors associated with the biological clock. DMSO was used as a control; other drugs used were Sirtinol, EX527, SR8278, and GSK4112. The effect of these drugs on the expression of *Per2* **(A)**, *ARNTL* (*Bmal*) **(B)**, *Reverb*-α **(C)**, *Per3* **(D)**, *ROR*-α **(E)**, and *Stra13* **(F)**.

For our purposes, cells at 5 days post differentiation were seeded at a density of 500,000 cells per well in a 6-well plate, then each well was treated overnight with 10 µM of the drug. After overnight culture, cells were collected for RNA extraction and qPCR analysis of the clock genes *Per2, ARNTL (Bmal), Rev-erb*-α, *Per3, ROR*-α, and *Stra13* was performed. As shown in Figure 5, Sirtinol and EX527 induced the expression of *Rev-erb*-α, *Per3*, and *ROR*-α, while only EX527 induced the expression of *Per2*. Sirtinol and EX527 repressed the expression of *ARNTL (Bmal)*, as shown in Figure 5B.

The *Rev-erb*-α ligand SR8278 either did not change the expression of *Per2, Rev-erb*-α, and *Per3*, or it repressed the expression of *ARNTL (Bmal)* and *ROR*-α. However, GSK4112 repressed all clock genes, with a greater effect on *Per2, Per3* and *ROR*-α.

As shown in Figure 5F, we also found that Sirtinol and EX527 increased the expression of *Stra13*, while the *Rev-erb*-α compounds SR8278 and GSK4112 did not change its expression.

### Effect of methylation and kinase pathway inhibitors on the expression of clock genes in P19 cells

**Figure 6:**
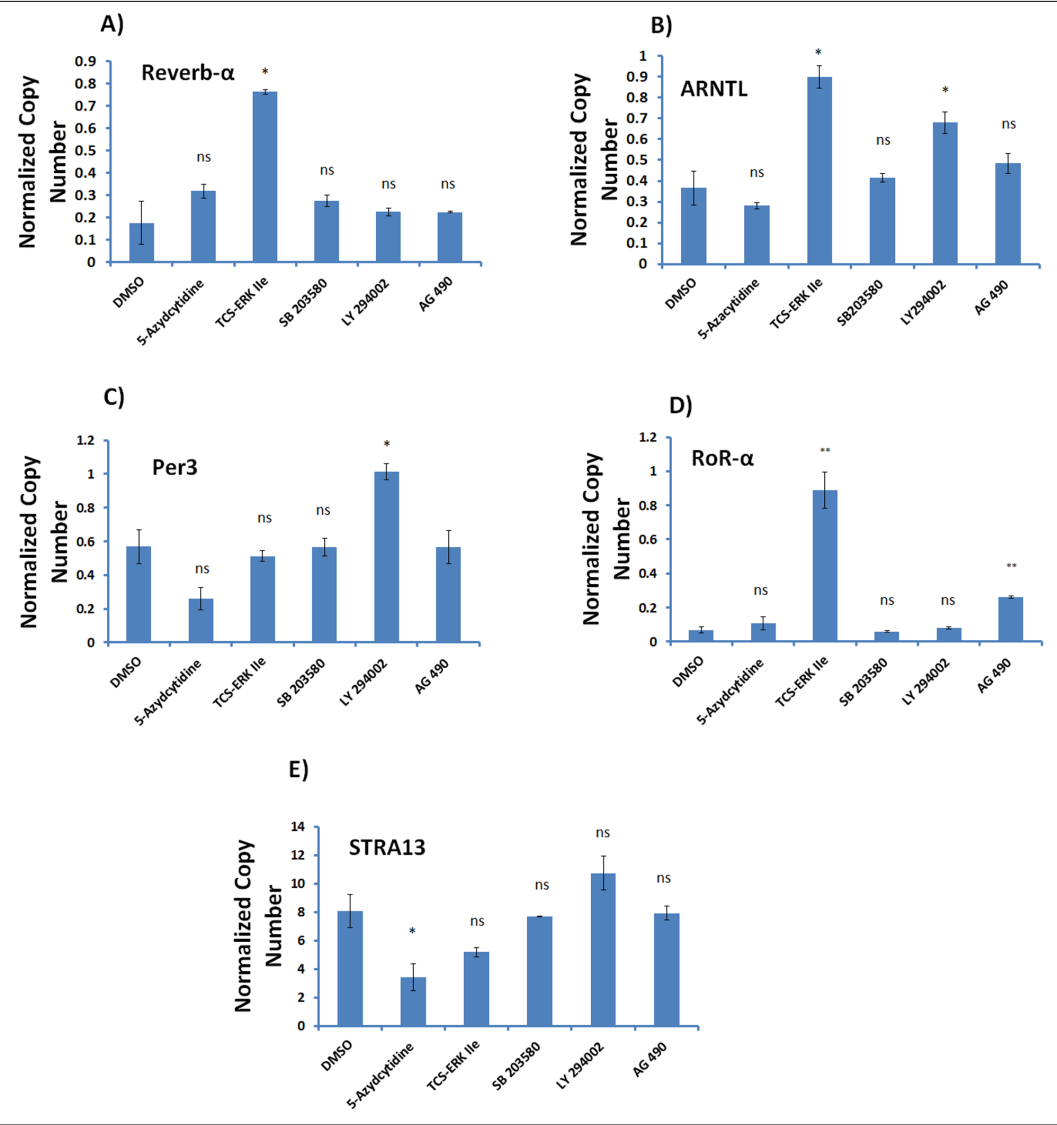
**Gene expression analysis of circadian clock genes in differentiated P19 cells in the presence of dimethylase and kinase inhibitors.** Differentiated P19 cells were seeded in a 6-well plate, then each well was treated with 10 μM of the indicated compound, or 1 μl of DMSO as control. After overnight culture, cells were collected for RNA extraction and qPCR analysis of the clock genes *Per2* **(A)**, *ARNTL* (*Bmal*) **(B)**, *Rev-erb*-α **(C)**, *Per3* **(D)**, *ROR*-α **(E)** and *Stra13* (F). Data shows that TCS-ERK IIe elevates the expression of *ARNTL* (*Bmal*) and *Rev-erb*-α, but has no effect on *ROR*-α. Expression of *Per3* and *ARNTL* (*Bmal*) increased more than *Rev-erb*-α and *ROR*-α, and by LY294002. 5-Azacytidine increased the expression of all clock genes at almost the same level.

Clock genes are all transcription factors regulated by epigenetic and kinase modulators [25,26,27]. Therefore, we decided to test compounds affecting these pathways on the expression of clock genes in differentiated P19 cells. In this regard, differentiated P19 cells were treated with 5-azacytidine, an inhibitor of DNA methylation; TCS-ERK IIe, an ERK inhibitor; SB203580, specific inhibitor of p38α and p38β; LY294002, a strong inhibitor of phosphoinositide 3-kinases (PI3Ks); and AG 460, and EGFR inhibitor. Cells were treated overnight at 10 µM final concentration, followed by gene expression analysis. As shown in Figure 6, the expression of all the examined genes increases with compound treatment. Compared to other compounds, AG490 has the most profound effect on the expression of *Per3, ARNTL (Bmal)* and *ROR*-α. However, the ERK inhibitor TCS-ERK IIe elevates the expression of *ARNTL (Bmal)* and *Rev-erb*-α, but does not significantly affect the expression of *ROR*-α and *Per3*. LY294002 also increases the expression of *Per3* and *ARNTL (Bmal)*, followed by *Rev-erb*-α and *ROR*-α.

5-azacytidine, a DNA methylation inhibitor, increased the expression of all clock genes to almost the same level as shown in Figure 6. This indicates that methylation is important in the regulation of the expression of these genes.

### Clock modulators increase neuronal differentiation markers in P19 cells

Since several compounds, including Sirtinol and kinase inhibitors, were found to modulate the expression of clock genes in differentiated P19 cells, we tested some of these to see whether they affected the differentiation of P19 cells. Selecting those compounds with the most significant effect on the expression of clock genes without causing cell death, we added these compounds during the cell aggregation and differentiation phases in the presence of 1 µM RA. Five days after differentiation treatment, we found that 2 µM Sirtinol caused an increase in the expression of *Hes5* and *Neurod1* by 3–4 times over RA treatment. Treatment with the ERK inhibitor TCS-ERK IIe did not change the expression of either gene in RA-treated cells. The *Rev-erb*-α agonist GSK4112, and the Sirt1 inhibitor Ex527 had no effect on the expression of *Neurod1*, but did inhibit the expression of *Hes5*.

## Discussion

Ever since the discovery of mammalian clock genes in 1994 [37], many biomedical and genetic studies have been carried out to explore the mammalian circadian rhythm [20]. However, while genetic studies have made a substantial contribution to our understanding and identification of clock components, there remains a considerable need for quantitative biochemical approaches to help scientists and researchers to further progress in this area [21,22]. A functional clock is present in adult stem cells. Here, circadian oscillations have a clear impact on driving proliferation and differentiation, thus enhancing both tissue homeostasis and regeneration [35]. Many in vitro studies have shown that pluripotent cells have no obvious rhythmicity, but their differentiated counterparts – even multipotent neural precursor cells (NPCs) – have robust circadian oscillations [36]. Yagita et al. (2010) demonstrated that these rhythms are lost upon reprogramming of these cells back to ES cells, and are regained upon redifferentiation to NPCs [8]. They concluded that cell-intrinsic rhythms of circadian gene expression are probably present at the very earliest stages of development.

It has been claimed that the lack of neurospherical rhythms in undifferentiated EC stem cells means that these cells would always fail to demonstrate any signs of circadian rhythms in their gene expression, unless they were allowed to differentiate by inducing them with a certain concentration of RA, as in the case of P19 cells [4]. Agreeing with this claim, our results show that the expression of clock genes changes over the course of differentiation (Figure 2), and treatment with RA leads to a decrease in the expression of most clock genes. This finding is important because it indirectly indicates that the clock genes are involved in the neuronal differentiation of P19 cells. However, contrary to the hypothesis that clock genes have no rhythmicity in undifferentiated stem cells, our results show for the first time that the expression of clock genes does indeed oscillate, both in undifferentiated and differentiated P19 cells. As shown in Figure 3, the oscillation was more pronounced for *Per3, ARNTL (Bmal), ROR*-α and *Rev-erb*-α genes. Using Biodare 2 software, the data were detrended to clearly show the clock gene oscillation (Figure 1, supplementary data). It is a novel finding that the clock oscillates in undifferentiated stem cells, and contradicts previous reports [4].

One hypothesis states that the clock does not oscillate, or that it is non-functional in stem cells to allow them to grow faster than differentiated cells [34,35,36,37]. Our findings suggest that oscillation of clock genes may be present in certain stem cells, and in future this information could be used as biomarkers to differentiate between different stem cell types. We induced oscillation using temperature change to synchronize the cells, rather than using a high percentage of FBS or dexamethasone, so as not to interfere with the induction of differentiation caused by RA in P19 cells.

The calculated period of *ARNTL (Bmal), Per2* and *Per3* ranged from 21.86–33.46 in undifferentiated and differentiated cells. These parameters are in the expected range [38], however the period seems to be longer in undifferentiated cells compared to differentiated P19 cells.

We looked into the expression of *Stra13*, an early RA-inducible gene, during the neuronal differentiation of P19 cells [16]. As expected, we found that *Stra13* is induced by RA (Figure 2F). Moreover, the expression of *Stra13* is dynamic, and changes at different stages of aggregation and differentiation during RA-induced neuronal differentiation of P19 cells (Figure 2F). This finding is interesting in itself because it indirectly indicates that the expression of *Stra13* is neatly regulated during the differentiation of P19 cells. *Stra13* is not only important for P19 differentiation, but its expression is also regulated by the molecular clock and biological rhythms. It has also been demonstrated that *Stra13* regulates a subset of peripheral clock genes, and its expression is rhythmically expressed in mouse peripheral organs that are regulated by the CLOCK–ARNTL heterodimer [17]. In our experiment, we examined the oscillation of *Stra13* gene expression in undifferentiated and differentiated P19 cells. As shown in Figure 3F, *Stra13* expression oscillates in differentiated P19 cells; this is consistent with earlier findings. The induction of *Stra13* by RA, and its oscillation, provides a solid biomarker to indicate that our experimental design was appropriate.

Importantly, we found that expression of the neuronal markers genes *Hes5* and *Tubb3*, but not *Neurod1*, oscillate in differentiated P19 cells (Figure 4). These results further strengthen the role of the molecular clock in neuronal differentiation, as described by Malik et al. (2015). However, the question arises: why does the expression of neuronal marker genes oscillate? One theory is that it is a mechanism by which the cells regulate their growth and function; perhaps these genes need to be switched on and off at different times of the day. This needs further investigation to understand its biological significance and role.

As reviewed by Masri [28] the molecular clock is not only regulated by the core transcriptional/translational feedback loop; it is also regulated by epigenetic modifications. For example, the histone methyltransferases MLL1 and MLL3 act on H3K4, which permits circadian gene expression. Intriguingly, the trimethylation of H3K4 is regulated by Sirt1 through the cyclic deacetylation of MLL1 [25,28]. Interestingly, the mammalian sirtuins regulate the circadian clock in the brain as well as peripheral clocks, such as in the liver. Sirt1 is involved in regulating circadian epigenetic control through the deacetylation of H3K9, modulation of *ARNTL (Bmal)*, the acetylation state and stability of *Per2*, and the subsequent control of circadian gene expression [29,30]. Bellet and colleagues [24] showed that activation of Sirt1 by small molecules induces the repression of circadian gene expression, and decreases H3K9/K14 acetylation at corresponding promoters in a time-specific manner. Specific activation of Sirt1 was demonstrated in vivo using liver-specific Sirt1-deficient mice; here the effect of Sirt1 activator compounds was shown to be dependent on Sirt1. The findings demonstrate that Sirt1 can fine-tune circadian rhythms, and pave the way to the development of pharmacological strategies to address a broad range of therapeutic indications [23]. In this regard, we tested the effect of small molecules that target Sirtuin, and also *Rev-erb*-α, on the expression of clock genes in differentiated P19 cells. As described in Figure 5, Sirtinol and EX527 induced the expression of *Rev-erb*-α, *Per3* and *ROR*-α, and at the same time repressed the expression of *ARNTL (Bmal)*. The expression of *Per2* was induced by EX527. The *Rev-erb*-α ligand SR8278 either did not change the expression, as in the case of *Per2, Rev-erb*-α, and *Per3*, or it repressed the expression, as for *ARNTL (Bmal)* and *ROR*-α. GSK4112 repressed all the clock genes.

We also found that Sirtinol and EX527 increased the expression of *Stra13*, while the *Rev-erb*-α compounds SR8278 and GSK4112 did not change its expression. The effect of Sirtuin and *Rev-erb*-α ligands on clock genes suggests that the circadian clock can be manipulated for therapeutic benefits. This finding also concurs with the earlier studies described above.

To identify other pathways that affect clock genes in P19 cells, we then tested demethylase and kinase inhibitors. Kinases, such as those in the mitogen-activated protein kinase (MAPK) pathways, can function as inputs, allowing the endogenous clock to entrain to a 24-hour environmental cycle. MAPK pathways can also interact physically and/or genetically with components of the molecular circadian oscillator, implying that MAPK pathways can affect the cycling of the clock [27].

We tested 5-azacytidine, an inhibitor of DNA methylation; TCS-ERK IIe, an ERK inhibitor; SB203580, a specific inhibitor of p38α and p38β; LY294002, a strong inhibitor of the phosphoinositide 3-kinases (PI3Ks); and AG460, an EGFR inhibitor. As described in Figure 6, the expression of all the genes increased after treatment with kinase compounds. Of the tested compounds, AG490 had the highest effect by substantially increasing the expression of *Per3, ARNTL (Bmal)* and *ROR*-α genes. 5-azacytidine, an inhibitor of DNA methylation, increased the expression of all the clock genes to almost the same level, as shown in Figure 6. This indicates that methylation is important in the regulation of the expression of these genes.

**Figure 7:**
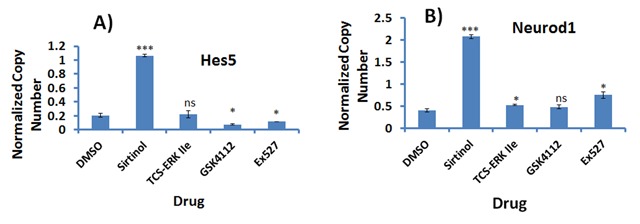
**Effect of clock gene-modulating compounds on neuronal differentiation of P19 embryonic stem cells.** P19 cells were differentiated using 1 μM retinoic acid (RA) for 5 days in the absence (DMSO) or presence of 2 μM final concentration of indicated compounds. After 5 days of differentiation, cells were collected for RNA extraction and qPCR analysis of the neuronal markers *Hes5* **(A)** and *Neurod1* **(B)**. The expression of *Hes5* and *Neurod1* increased by 3–4 times when treated with 2 μM Sirtinol during differentiation, while TCS-ERK IIe changed the expression of either gene in cells.

As we found that Sirtuin, *Rev-erb*-α and ERK inhibitors have the most striking effect on the expression of clock genes, we then tested these ligands on the neuronal differentiation of P19 cells. As shown in Figure 7, the expression of *Hes5* and *Neurod1* increased by 3–4 times compared to treatment with RA when Sirtinol (Sirt1 and Sirt2) was added during differentiation at a final concentration of 2 µM. Treatment with the ERK inhibitor TCS-ERK IIe did not change the expression of either gene over RA-treated cells. As found in previous studies, neither the *Rev-erb*-α agonist GSK4112, nor the Sirt1 inhibitor Ex527, changed the expression of *Neurod1*, but they did inhibit the expression of *Hes5*.

The commitment and differentiation of multipotent neural stem cells (NSCs) are tightly controlled by intrinsic and extrinsic regulatory mechanisms in space and time-related manners. As reviewed in Cai et al. (2016), Sirt1 is expressed in several areas of the brain, and has been reported to be involved in self-renewal, multipotency, and fate determination of NSCs. Recent studies have highlighted the role of the deacetylase activity of Sirt1 in the determination of the final fate of NSCs [31]. It has also been shown that Sirt2 regulates neuronal differentiation through the ERK/CREB pathway [32]. While the mechanism of this action is not yet known, one study led by Heo et al. (2017) demonstrated that Sirt1 regulates DNA methylation by antagonizing the transcription and protein stability of Dnmt3l, thereby preserving the proper developmental potency of ESCs [39].

Numerous studies have demonstrated a direct or indirect link between kinase pathways that include ERK, PI3K and AKT in neuronal differentiation. They are also involved in the differentiation of embryonic neural stem/progenitor cells (eNSPCs), during which most kinase pathways are activated [33,34].

In conclusion, our study is the first to show that the expression of clock genes oscillates in both undifferentiated and differentiated P19 stem cells, and that expression of the differentiation and neuronal marker genes *Hes5, Tubb3* and *Stra13* is rhythmic. The expression of all of these genes is sensitive to treatment with small molecules.

The oscillation of the clock in undifferentiated P19 cells might be attributed to the nature of these cancerous stem cells, which are prone to differentiate into neurons and cardiomyocytes; they are distinct from normal stem cells that are able to differentiate into multiple lineages. In this regard, P19 stem cells may already be committed to differentiate into neurons and cardiomyocytes, and this may affect their clock biology. Our data could be used to assess whether other stem cells are pure, uncommitted stem cells (that is, if their clock does not oscillate), or already committed stem cells, such as P19 cells, which have an oscillating biological clock. Our findings may open a new chapter in the clock biology of stem cells, and how it can be modulated to direct the differentiation of stem cells into different lineages.

## Additional File

The additional file for this article can be found as follows:

10.5334/jcr.157.s1 Supplementary Figure 1 Detrended Oscillation and Clock Parameters.
