# Supplementary material for: P19 Cells as a Model for Studying the Circadian Clock in Stem Cells before and after Cell Differentiation
Source: J Circadian Rhythms. 2018 May 18;16:6. doi: 10.5334/jcr.157 (PMC6083773; doi:10.5334/jcr.157)
Supplement: Supplementary Figure 1 — Detrended Oscillation and Clock Parameters. [file jcr-16-157-s1.pdf]

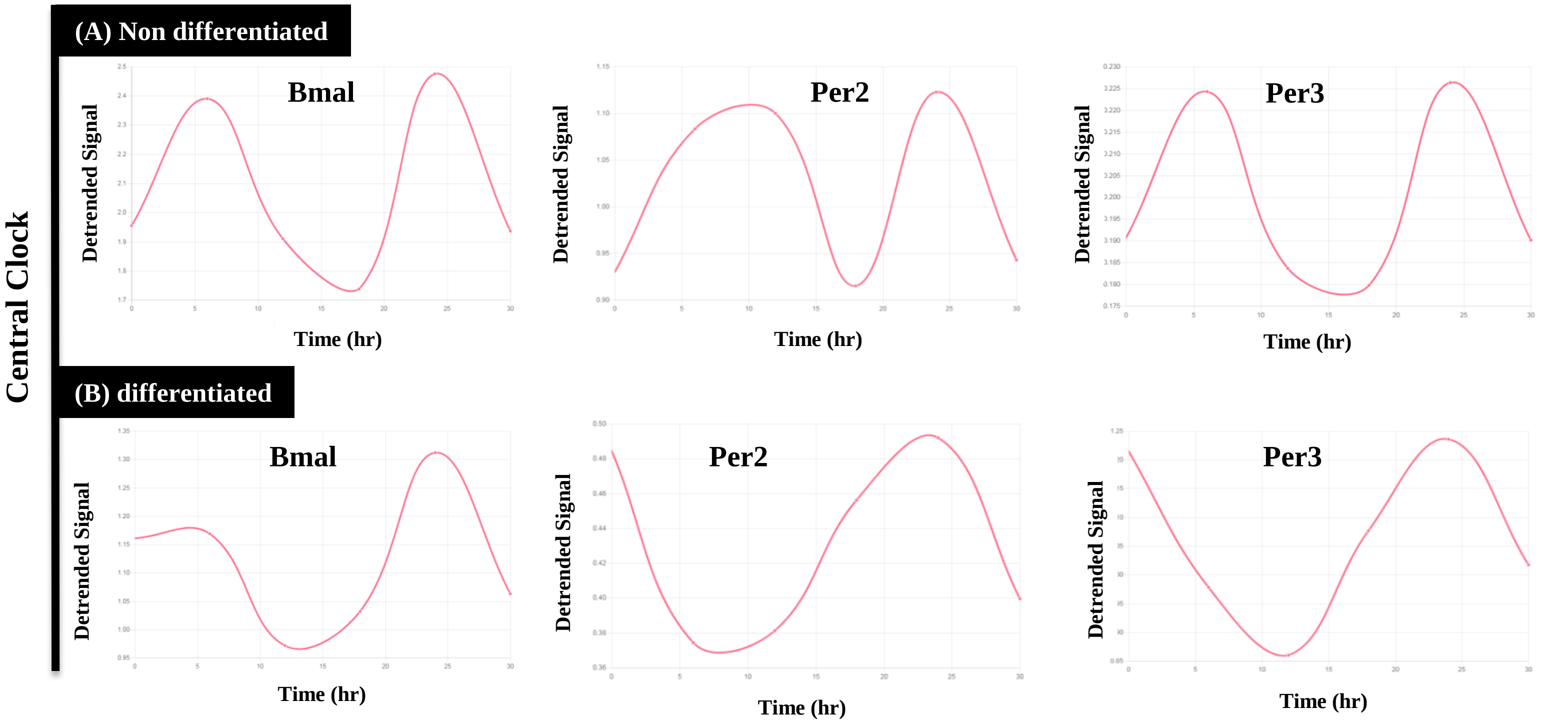

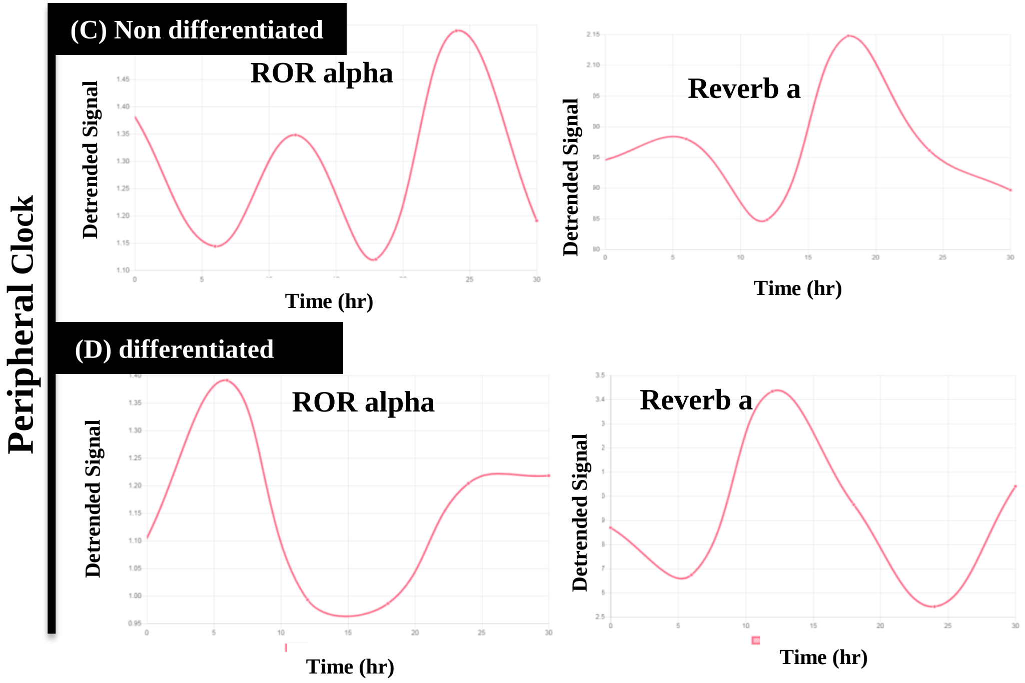

(E) Clock Parameters

	Non Differentiated			Differentiated		
	Period (hr)	Phase (hr)	Amp.	Period (hr)	Phase (hr)	Amp.
Bmal	33.46	19.83	5.0E-02	20.22	4.49	1.6E-01
Per2	31.12	23.42	6.0E-02	22.04	23.12	6.0E-02
Per3	21.86	7.36	6.0E-02	22.98	23.96	1.8E-01
Reverb a	19.74	16.63	4.1E-01	22.6	20.42	8.0E-02
ROR a	19.9	4.27	8.0E-02	31.18	23.3	4.0E-02

Supplementary Figure 1: Detrended Oscillation and Clock Parameters

(A-D) The data of Figure xx of oscillation of central and peripheral clock genes in non-differentiated and differentiated P19 cells were subjected to detrending using online clock software Biodare 2.The detrending was performed using Linear detrending, which is the least intrusive detrending method.

(E) The Clock parameters , Period, Phase and Amplitude of non-differentiated and differentiated P19 cells were calculated using the Morfitt that generally gives the most accurate period estimates from all the methods used (38).
